# Thalamic nuclei volumes and intrinsic thalamic network in patients with occipital lobe epilepsy

**DOI:** 10.1002/brb3.2968

**Published:** 2023-03-16

**Authors:** Dong Ah Lee, Ho‐Joon Lee, Kang Min Park

**Affiliations:** ^1^ Department of Neurology Haeundae Paik Hospital, Inje University College of Medicine Busan Republic of Korea; ^2^ Department of Radiology Haeundae Paik Hospital, Inje University College of Medicine Busan Republic of Korea

**Keywords:** epilepsies, magnetic resonance imaging, occipital lobe, thalamus

## Abstract

**Introduction:**

This study aimed to investigate the alterations in individual thalamic nuclei volumes in patients with occipital lobe epilepsy (OLE) compared with those of healthy controls, and to analyze the intrinsic thalamic network based on these volumes using graph theory.

**Methods:**

Thirty adult patients with newly diagnosed OLE and 42 healthy controls were retrospectively enrolled (mean age, 33.8 ± 17.0 and 32.2 ± 6.6 years, respectively). The study participants underwent brain magnetic resonance imaging with three‐dimensional T1‐weighted imaging. The right and left total thalamic and individual thalamic nuclei volumes were obtained using the FreeSurfer program. Then, the intrinsic thalamic network was calculated based on the individual thalamic nuclei volumes and graph theory using a BRAPH program.

**Results:**

There were no differences in the right and left whole‐thalamic volumes between the two groups (0.445% vs. 0.469%, *p* = .142 and 0.481% vs. 0.490%, *p* = .575, respectively). However, significant differences were observed in the volumes of several thalamic nuclei between the two groups. The right medial geniculate and right suprageniculate nuclei volumes were increased (0.0077% vs. 0.0064%, *p* = .0003 and 0.0013% vs. 0.0010%, *p* = .0004, respectively), whereas the right and left parafascicular nuclei volumes were decreased in patients with OLE compared with those in healthy controls (0.0038% vs. 0.0048%, *p* < .0001 and 0.0037% vs. 0.0045%, *p* = .0001, respectively). There were no differences in the network measures regarding intrinsic thalamic network between the two groups.

**Conclusion:**

We successfully demonstrated the alterations in individual thalamic nuclei volumes, especially the increased medial geniculate and suprageniculate, and decreased parafascicular nuclei volumes in patients with OLE compared with those of healthy controls despite no changes in the whole‐thalamic volumes. These findings suggest an important role of the thalamus in the epileptic network of OLE.

## Graphical abstract

Alterations in individual thalamic nuclei volumes can be observed in patients with OLE.

Some individual thalamic nuclei volumes were correlated with clinical characteristics in patients with OLE.

Thalamus plays an important role in the epileptic network of OLE.

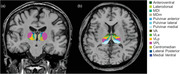

## 1 INTRODUCTION

Occipital lobe epilepsy (OLE) represents a minor percentage of epilepsy and accounts for approximately 5% of focal epilepsies (Adcock & Panayiotopoulos, 2012; Angus‐Leppan & Clay, 2021). The initial ictal symptoms of OLE include elementary visual hallucinations, visual illusions, ictal blindness, eye blinking, ictal nystagmus, and ocular deviation (Adcock & Panayiotopoulos, 2012; Angus‐Leppan & Clay, 2021). Seizure propagation from the occipital lobe to the frontal lobe or neighboring temporal and parietal lobes, as well as the midbrain tegmentum, makes it difficult to delineate the seizure‐onset zone in OLE (Heo et al., 2018). Thus, it is often difficult to determine whether seizures associated with visual aura originate from the occipital or temporal lobes, although electroencephalography (EEG) can help differentiate between the two epilepsies (Appel et al., 2015).

As the concept of network disease in epilepsy has been recently established by several researches, not only the cortex, which is traditionally considered the origin of epilepsy, but also subcortical structures have recently received attention (Park et al., 2019). Of the several subcortical structures, in particular, thalamus, previously known to play an important role in the spike‐and‐slow‐wave complexes observed in generalized epilepsy, has been found to be involved in the epileptic brain network in focal epilepsy as well (Cho et al., 2021; Lee & Park, 2020; Lee, Seo, & Park, 2020; Lee, Seo, Lee, et al., 2020). Volumetric analysis using brain magnetic resonance imaging (MRI) has revealed that patients with generalized epilepsy have a significant decrease in gray matter volumes in the bilateral thalamus, which is also negatively correlated with the duration of epilepsy (Huang et al., 2011). Furthermore, a previous study in patients with temporal lobe epilepsy showed a decrease in thalamic nuclei volumes, especially the parafascicular nucleus, and revealed that the changes in thalamic nuclei volumes are dependent on the response to antiseizure medications (ASMs) (Lee, Seo, & Park, 2020). In addition, a study on patients with temporal lobe epilepsy analyzed the intrinsic thalamic network using these thalamic nuclei volumes and graph theory, and demonstrated that the preoperative intrinsic thalamic network is related to postsurgical outcomes (Cho et al., 2021). Additionally, significant volume reductions in the right and left thalamus have been noted in patients with frontal lobe epilepsy (Rahatli et al., 2020). However, no study has focused on the thalamic changes in patients with OLE, and no research has been conducted to investigate the alterations in individual thalamic nuclei volumes and intrinsic thalamic network based on these volumes and graph theory. Considering the rapid seizure propagation to other lobes in patients with OLE, it is likely that the thalamus plays an important role in this epileptic network. Thus, structural changes in the thalamus can be expected in patients with OLE.

This study investigated the individual thalamic nuclei volumes in patients with OLE and healthy controls, and calculated the intrinsic thalamic network based on these volumes using graph theoretical analysis. Then, we compared the thalamic nuclei volumes and intrinsic thalamic network between the patients with OLE and healthy controls. We hypothesized that there were significant differences in the thalamic nuclei volumes or intrinsic thalamic network between the two groups.

## 2 METHODS

### 2.1 Participants

**FIGURE 1:**
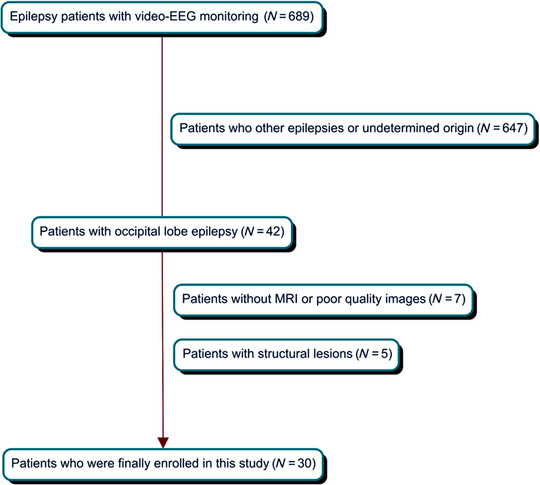
The selection process for enrollment of patients with occipital lobe epilepsy in this study.

The present study was approved by the Institutional Review Board of our hospital. This research included a retrospective study performed at a tertiary hospital. From the database of the video‐EEG monitoring unit at our epilepsy center from March 2010 to May 2022, we enrolled 30 patients with newly diagnosed OLE, who met the following inclusion criteria: (1) seizure semiology typical for OLE (Angus‐Leppan & Clay, 2021; Sveinbjornsdottir & Duncan, 1993), (2) ictal EEG originating from the occipital lobe, and (3) three‐dimensional T1‐weighted imaging at the time of epilepsy diagnosis with drug‐naïve status. We excluded individuals with any other visible MRI abnormalities or any medical or neurological disease history except epilepsy (Figure 1). Each patient's age, sex, age at seizure onset, and ictal semiology were recorded.

Additionally, we recruited 42 healthy, age‐ and sex‐matched controls with no history of any medical or neurological disorders. They had been already recruited for our previous study, which had enrolled 150 healthy participants (Jang et al., 2017). Of the 150 patients, those who did not consent to the use of their data in this study were excluded, and the remaining healthy participants with matched age and sex as the patients with OLE were included in the control group. All healthy controls had normal brain MRI.

### 2.2 Brain MRI acquisition

Patients with OLE and healthy controls underwent brain MRI using the same sequences on the same 32‐channel head coil‐equipped 3‐Tesla MRI scanner (AchievaTx, Phillips Healthcare). Three‐dimensional fluid‐attenuated inversion recovery, coronal T2‐weighted imaging, and three‐dimensional T1‐weighted imaging were also included among the MR sequences to exclude the structural lesions. The T1‐weighted images were acquired using a turbo‐field echo sequence with the following parameters: inversion time = 1300 ms, repetition time/echo time = 8.6/3.96 ms, flip angle = 8°, and voxel size of 1 mm^3^.

### 2.3 Calculation of thalamic nuclei volume

**FIGURE 2:**
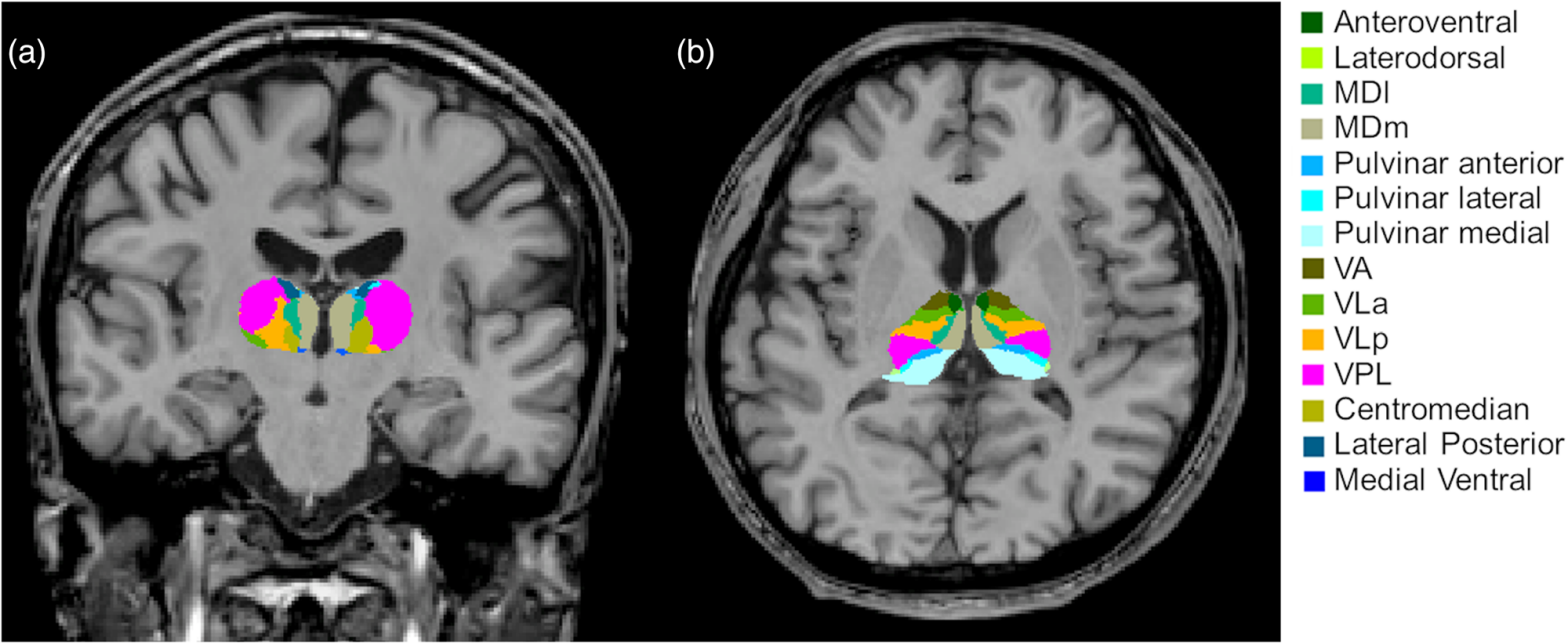
Example of thalamic nuclei segmentation in a patient with occipital lobe epilepsy. Segmentations and labels of thalamic nuclei in the coronal (a) and axial (b) planes generated by FreeSurfer (not all segmentations are shown). The segmentations are overlaid on the T1‐weighted scan. The figure has been generated from our previous study (Shin et al., 2019). MDl, mediodorsal lateral parvocellular nucleus; MDm, mediodorsal medial magnocellular nucleus; VLa, ventral lateral anterior nucleus; VLp, ventral lateral posterior nucleus; VPL, ventral posterolateral nucleus; and VA, ventral anterior nucleus.

Previously, we described the method for calculating the total thalamic and individual thalamic nuclei volumes using FreeSurfer version 7.0.21 (Shin et al., 2019). Briefly, using the “recon‐all” and “segmentThalamicNuclei.sh” functions, the right and left whole‐thalamic volumes and individual thalamic nuclei volumes were automatically obtained (Iglesias et al., 2018). Figure 2 displays an example of segmentation of thalamic nuclei (Shin et al., 2019). Individual thalamic nuclei included the right and left anteroventral nuclei in the anterior group; right and left laterodorsal and lateral posterior nuclei in the lateral group; right and left ventral anterior, ventral anterior magnocellular, ventral lateral anterior, ventral lateral posterior, ventral posterolateral, and ventromedial nuclei in the ventral group; right and left central medial, central lateral, paracentral, centromedian, and parafascicular nuclei in the intralaminar group; right and left paratenial, medial ventral, mediodorsal medial magnocellular, and mediodorsal lateral parvocellular nuclei in the medial group; and right and left lateral geniculate, medial geniculate, suprageniculate, pulvinar anterior, pulvinar medial, pulvinar lateral, and pulvinar inferior nuclei in the posterior group. Then, we adjusted the whole‐thalamic volumes and individual thalamic nuclei volumes using the estimated total intracranial volume.

### 2.4 Calculation of the intrinsic thalamic network

To obtain the intrinsic thalamic network, we employed a graph theoretical analysis. Using a BRAPH program (Mijalkov et al., 2017), we created a weighted connectivity matrix for patients with OLE and healthy controls, using nodes and edges, respectively. We defined nodes as individual thalamic nuclei volumes, and edges as partial correlation between the volumes with controlling for the effects of age and sex. We extracted the network measures, such as average degree, average strength, radius, diameter, eccentricity, characteristic path length, global efficiency, local efficiency, mean clustering coefficient, transitivity, modularity, assortative coefficient, and small‐worldness index from the connectivity matrix (Falsaperla et al., 2021; Farahani et al., 2019; Mijalkov et al., 2017; Park et al., 2019; Schrodt et al., 2020; Zenil et al., 2018).

### 2.5 Statistical analysis

No statistical power calculations were performed prior to the study. The sample size was based on the available data. We utilized the Student's *t*‐test for age and volume comparisons, and the Fisher's exact test for sex comparisons between groups. Correlation analysis was performed using the Pearson's method. All statistical analyses were conducted using MedCalc^®^ (MedCalc Software version 20.014, Ostend, Belgium; http://www.medcalc.org; 2020). Nonparametric permutation tests with 1000 permutations were used to compare network measures as we could obtain group‐level network measures. The permutation test was conducted directly within the BRAPH application. Statistical significance was set at *p*‐value <.05. In the analysis of group differences in terms of thalamic nuclei volumes, the *p*‐value was set with multiple corrections with Bonferroni method (*p* = .05/50 numbers of the thalamic nuclei = .001).

## 3 RESULTS

### 3.1 Clinical characteristics of patients with OLE

Table 1 shows the clinical characteristics in the patients with OLE and healthy controls. Of the 30 patients with epilepsy, 16 patients were right OLE, whereas 13 patients had left OLE. One patient was difficult to lateralize the seizure origin. Age and sex were not different between the patients with OLE and healthy controls (33.8 vs. 32.2 years, *p* = .552 and 12/30 (50%) vs. 21/42 (50%), *p* = 1.000, respectively).

**TABLE 1:** Clinical characteristics in patients with occipital lobe epilepsy

	Patients with occipital lobe epilepsy (*N* = 30)	Healthy controls (*N* = 42)	*p*
Age ± SD, years	33.8 ± 17.0	32.2 ± 6.6	.552
Male gender, *N* (%)	15 (50.0)	21 (50.0)	1.000
Age of seizure onset, years (interquartile range)	24 (18.2–31.3)		
Initial seizure semiology			
Visual symptoms, *N* (%)	16 (53.3)		
Oculomotor, *N* (%)	10 (33.3)		
Others, *N* (%)	4 (13.3)		
Epileptiform occipital activity on EEG, N (%)	30 (100)		

Abbreviations: EEG, electroencephalography; SD, standard deviation.

### 3.2 Differences in the thalamic nucleus volumes between patients with OLE and healthy controls

Table 2 shows the difference in the thalamic nuclei volumes between the patients with OLE and healthy controls. There were no differences in the right and left whole‐thalamic volumes between the two groups (0.445% vs. 0.469%, *p* = .142 and 0.481% vs. 0.490%, *p* = .575, respectively). However, several thalamic nuclei showed significant difference in volumes between the patients with OLE and healthy controls. The right medial geniculate and right suprageniculate nuclei volumes were increased (0.0077% vs. 0.0064%, *p* = .0003 and 0.0013% vs. 0.0010%, *p* = .0004, respectively), whereas the right and left parafascicular nuclei volumes were decreased in patients with OLE compared with those in healthy controls (0.0038% vs. 0.0048%, *p* < .0001 and 0.0037% vs. 0.0045%, *p* = .0001, respectively).

**TABLE 2:** Differences of the thalamic nuclei volumes between patients with occipital lobe epilepsy and healthy controls

		Patients with occipital lobe epilepsy (*N* = 30)		Healthy controls (*N* = 42)			
		Mean (%)	SD (%)	Mean (%)	SD (%)	Difference	*p*
Right whole thalamus		0.4459	0.08351	0.4693	0.05013	0.02344	.1421
Left whole thalamus		0.4817	0.07675	0.4903	0.05267	0.00859	.575
Right thalamic group	Nucleus						
Anterior	Anteroventral	0.00839	0.00269	0.00899	0.00149	0.00061	.2252
Lateral	Laterodorsal	0.00147	0.00072	0.00165	0.00057	0.00019	.2244
	Lateral posterior	0.00765	0.00219	0.00841	0.00124	0.00076	.066
Ventral	Ventral anterior	0.02615	0.00674	0.02711	0.00298	0.00096	.413
	Ventral anterior magnocellular	0.00228	0.00053	0.00241	0.00032	0.00013	.2028
	Ventral lateral anterior	0.04264	0.00972	0.0458	0.00504	0.00316	.0765
	Ventral lateral posterior	0.05656	0.01141	0.06134	0.00721	0.00478	.0329
	Ventral posterolateral	0.06452	0.01228	0.06936	0.00928	0.00485	.0603
	Ventromedial	0.00181	0.0005	0.00213	0.00031	0.00032	.0013
Intralaminar	Central medial	0.00451	0.00144	0.00479	0.00069	0.00029	.2587
	Central lateral	0.00235	0.0007	0.00243	0.00055	0.00008	.5889
	Paracentral	0.00028	0.00009	0.00031	0.00004	0.00003	.0393
	Centromedian	0.01714	0.00306	0.0187	0.00226	0.00156	.0152
	Parafascicular	0.00389	0.00097	0.00488	0.00073	0.00099	<.0001*
Medial	Paratenial	0.0005	0.00008	0.00051	0.00007	0.00001	.6746
	Medial ventral	0.00082	0.00034	0.00088	0.00017	0.00007	.278
	Mediodorsal medial magnocellular	0.05268	0.01179	0.05182	0.00755	−0.00086	.7056
	Mediodorsal lateral parvocellular	0.01772	0.00374	0.01765	0.00269	−0.00008	.9208
Posterior	Lateral geniculate	0.01672	0.00451	0.01887	0.00252	0.00216	.0118
	Medial geniculate	0.00777	0.00181	0.00643	0.00117	−0.00134	.0003*
	Suprageniculate	0.00133	0.00046	0.00103	0.00021	−0.0003	.0004*
	Pulvinar anterior	0.01341	0.00273	0.01432	0.00177	0.0009	.0933
	Pulvinar medial	0.07013	0.01199	0.07244	0.00809	0.00231	.3315
	Pulvinar lateral	0.01068	0.0015	0.01121	0.00165	0.00054	.1629
	Pulvinar inferior	0.01448	0.00289	0.01581	0.00217	0.00134	.028
Left thalamic group	Nucleus						
Anterior	Anteroventral	0.0087	0.00158	0.00869	0.00114	−0.00001	.9735
Lateral	Laterodorsal	0.00156	0.00072	0.00168	0.00047	0.00012	.4017
	Lateral posterior	0.00815	0.00204	0.00866	0.00141	0.00051	.2115
Ventral	Ventral anterior	0.02814	0.0042	0.02696	0.00276	−0.00118	.1535
	Ventral anterior magnocellular	0.00238	0.00041	0.00233	0.00031	−0.00005	.5569
	Ventral lateral anterior	0.0461	0.00734	0.04615	0.00507	0.00006	.9693
	Ventral lateral posterior	0.06169	0.01053	0.06408	0.00762	0.00239	.2673
	Ventral posterolateral	0.07026	0.01313	0.07448	0.00976	0.00422	.1225
	Ventromedial	0.00186	0.0004	0.00209	0.00029	0.00023	.0061
Intralaminar	Central medial	0.0047	0.00102	0.00476	0.00073	0.00006	.7783
	Central lateral	0.00235	0.00057	0.00231	0.00044	−0.00004	.7513
	Paracentral	0.00028	0.00005	0.00028	0.00004	0	.883
	Centromedian	0.01716	0.0026	0.01845	0.00233	0.00129	.0309
	Parafascicular	0.00375	0.00072	0.00458	0.00089	0.00083	.0001*
Medial	Paratenial	0.0005	0.00009	0.00048	0.00006	−0.00002	.2438
	Medial ventral	0.00083	0.00029	0.00086	0.00017	0.00003	.5798
	Mediodorsal medial magnocellular	0.05608	0.01084	0.05328	0.00738	−0.00279	.1976
	Mediodorsal lateral parvocellular	0.01847	0.0034	0.01795	0.00257	−0.00052	.4586
Posterior	Lateral geniculate	0.01858	0.00476	0.01944	0.00256	0.00085	.33
	Medial geniculate	0.00646	0.00171	0.0054	0.00111	−0.00106	.002
	Suprageniculate	0.00122	0.00034	0.00103	0.0004	−0.00019	.0399
	Pulvinar anterior	0.01552	0.00252	0.01596	0.002	0.00045	.4052
	Pulvinar medial	0.07725	0.01326	0.07851	0.00907	0.00126	.6323
	Pulvinar lateral	0.01283	0.00238	0.01374	0.00259	0.00092	.1308
	Pulvinar inferior	0.01687	0.00376	0.01812	0.00236	0.00125	.0871

Abbreviation: SD, standard deviation.

* *p* < .001.

### 3.3 Differences in the intrinsic thalamic network between patients with OLE and healthy controls

Table 3 shows the differences in the intrinsic thalamic network between the patients with OLE and healthy controls. No significant differences were observed between the two groups in terms of network measures regarding intrinsic thalamic network, including average degree, average strength, radius, diameter, eccentricity, characteristic path length, global efficiency, local efficiency, mean clustering coefficient, transitivity, modularity, assortative coefficient, and small‐worldness index.

**TABLE 3:** Differences of the intrinsic thalamic network between patients with occipital lobe epilepsy and healthy controls

	Patients with occipital lobe epilepsy (*N* = 30)	Healthy control (*N* = 42)	Difference	CI lower	CI upper	*p*
Average degree	48.800	48.640	−0.160	−1.157	2.803	.318
Average strength	28.580	25.965	−2.616	−7.146	7.148	.274
Radius	2.692	2.467	−0.226	−0.894	0.947	.330
Diameter	4.416	4.527	0.111	−1.923	1.802	.457
Eccentricity	3.438	3.649	0.210	−1.267	1.219	.402
Characteristic path length	1.852	2.055	0.203	−0.577	0.565	.281
Global efficiency	0.596	0.543	−0.053	−0.124	0.125	.269
Local efficiency	2.532	2.106	−0.426	−1.022	0.925	.283
Mean clustering coefficient	0.565	0.512	−0.052	−0.144	0.147	.286
Transitivity	0.848	0.771	−0.077	−0.218	0.221	.292
Modularity	0.042	0.038	−0.004	−0.050	0.037	.465
Assortative coefficient	−0.027	−0.038	−0.011	−0.028	0.034	.255
Small‐worldness index	0.970	0.966	−0.004	−0.037	0.045	.380

Abbreviations: CI, 95% confidence interval of difference between the groups.

### 3.4 Correlation analysis between the clinical characteristics and thalamic nuclei volumes in patients with OLE

Table 4 shows the correlation analysis between the clinical characteristics and the total thalamic and individual thalamic nuclei volumes in patients with OLE. Age significantly correlated with the right mediodorsal medial magnocellular, right mediodorsal lateral parvocellular, and right pulvinar medial nuclei volumes (*r* = −.389, *p* = .037; *r* = −.380, *p* = .042; and *r* = −.550, *p* = .002, respectively), and age at seizure onset correlated with the right pulvinar anterior and right pulvinar medial nuclei volumes (*r* = −.519, *p* = .023 and *r* = −.471, *p* = .042, respectively).

**TABLE 4:** Correlation analysis between clinical characteristics and thalamic nuclei volumes in patients with occipital lobe epilepsy

			Age	Age at seizure onset
Right whole thalamus		Correlation coefficient	−.280	−.315
		*p*‐value	.141	.189
Left whole thalamus		Correlation coefficient	−.024	−.179
		*p*‐value	.900	.463
Right thalamic group	Nucleus			
Anterior	Anteroventral	Correlation coefficient	−.065	−.068
		*p*‐value	.737	.783
Lateral	Laterodorsal	Correlation coefficient	−.010	.006
		*p*‐value	.960	.980
	Lateral posterior	Correlation coefficient	−.017	.023
		*p*‐value	.930	.925
Ventral	Ventral anterior	Correlation coefficient	−.311	−.177
		*p*‐value	.101	.468
	Ventral anterior magnocellular	Correlation coefficient	−.320	−.259
		*p*‐value	.090	.285
	Ventral lateral anterior	Correlation coefficient	−.300	−.202
		*p*‐value	.114	.408
	Ventral lateral posterior	Correlation coefficient	−.242	−.204
		*p*‐value	.207	.403
	Ventral posterolateral	Correlation coefficient	−.112	−.137
		*p*‐value	.562	.576
	Ventromedial	Correlation coefficient	−.003	−.043
		*p*‐value	.986	.862
Intralaminar	Central medial	Correlation coefficient	−.249	−.156
		*p*‐value	.193	.524
	Central lateral	Correlation coefficient	.164	.001
		*p*‐value	.396	.996
	Paracentral	Correlation coefficient	−.280	−.094
		*p*‐value	.141	.701
	Centromedian	Correlation coefficient	.122	−.078
		*p*‐value	.529	.752
	Parafascicular	Correlation coefficient	.105	.021
		*p*‐value	.588	.933
Medial	Paratenial	Correlation coefficient	.082	−.065
		*p*‐value	.674	.791
	Medial ventral	Correlation coefficient	−.216	−.228
		*p*‐value	.259	.348
	Mediodorsal medial magnocellular	Correlation coefficient	−.389	−.432
		*p*‐value	.037*	.065
	Mediodorsal lateral parvocellular	Correlation coefficient	−.380	−.438
		*p*‐value	.042*	.061
Posterior	Lateral geniculate	Correlation coefficient	−.282	−.235
		*p*‐value	.138	.333
	Medial geniculate	Correlation coefficient	.073	−.100
		*p*‐value	.709	.684
	Suprageniculate	Correlation coefficient	.087	.144
		*p*‐value	.653	.556
	Pulvinar anterior	Correlation coefficient	−.550	−.519
		*p*‐value	.002*	.023*
	Pulvinar medial	Correlation coefficient	−.314	−.471
		*p*‐value	.097	.042*
	Pulvinar lateral	Correlation coefficient	−.334	−.363
		*p*‐value	.077	.127
	Pulvinar inferior	Correlation coefficient	−.356	−.432
		*p*‐value	.058	.065
Left thalamic group	Nucleus			
Anterior	Anteroventral	Correlation coefficient	.043	−.061
		*p*‐value	.824	.805
Lateral	Laterodorsal	Correlation coefficient	−.135	−.060
		*p*‐value	.485	.806
	Lateral posterior	Correlation coefficient	.014	−.002
		*p*‐value	.945	.994
Ventral	Ventral anterior	Correlation coefficient	−.222	−.277
		*p*‐value	.248	.251
	Ventral anterior magnocellular	Correlation coefficient	−.196	−.282
		*p*‐value	.309	.242
	Ventral lateral anterior	Correlation coefficient	−.104	−.261
		*p*‐value	.593	.280
	Ventral lateral posterior	Correlation coefficient	−.006	−.222
		*p*‐value	.975	.360
	Ventral posterolateral	Correlation coefficient	.153	−.065
		*p*‐value	.429	.790
	Ventromedial	Correlation coefficient	.153	−.091
		*p*‐value	.429	.712
Intralaminar	Central medial	Correlation coefficient	−.069	−.114
		*p*‐value	.720	.641
	Central lateral	Correlation coefficient	.215	.035
		*p*‐value	.263	.886
	Paracentral	Correlation coefficient	−.162	−.231
		*p*‐value	.400	.341
	Centromedian	Correlation coefficient	.188	−.156
		*p*‐value	.328	.523
	Parafascicular	Correlation coefficient	.213	.117
		*p*‐value	.266	.634
Medial	Paratenial	Correlation coefficient	.172	−.173
		*p*‐value	.373	.479
	Medial ventral	Correlation coefficient	−.123	−.099
		*p*‐value	.526	.686
	Mediodorsal medial magnocellular	Correlation coefficient	−.240	−.295
		*p*‐value	.210	.220
	Mediodorsal lateral parvocellular	Correlation coefficient	−.210	−.299
		*p*‐value	.275	.213
Posterior	Lateral geniculate	Correlation coefficient	.082	−.093
		*p*‐value	.671	.706
	Medial geniculate	Correlation coefficient	.001	−.273
		*p*‐value	.997	.258
	Suprageniculate	Correlation coefficient	−.010	−.207
		*p*‐value	.961	.395
	Pulvinar anterior	Correlation coefficient	−.180	−.150
		*p*‐value	.350	.540
	Pulvinar medial	Correlation coefficient	.030	−.038
		*p*‐value	.875	.877
	Pulvinar lateral	Correlation coefficient	−.006	.010
		*p*‐value	.975	.969
	Pulvinar inferior	Correlation coefficient	.015	−.017
		*p*‐value	.939	.944

* *p* < .05.

## 4 DISCUSSION

The main findings of this study were significant differences in the volumes of individual thalamic nuclei, especially the increased medial geniculate and suprageniculate, and decreased parafascicular nuclei volumes in patients with OLE compared with those of healthy controls despite no changes in the whole‐thalamic volumes. In addition, in patients with OLE, some of the individual thalamic nuclei volumes were well correlated with clinical characteristics, including age and age at seizure onset. However, we found no differences in the intrinsic thalamic network between the patients with OLE and healthy controls.

Our study revealed that the medial geniculate and suprageniculate nuclei volumes were increased in patients with OLE compared with those in healthy controls. The medial geniculate nucleus in the thalamus is located medially and caudally to the considerably larger lateral geniculate nucleus (Calford & Aitkin, 1983; Rouiller et al., 1985). The medial geniculate nucleus is the auditory relay center of the thalamus that receives auditory data from the inferior colliculus. Conversely, the primary auditory cortex, also known as the transverse temporal gyri of Heschl, and the auditory association cortex receive output from the medial geniculate nucleus, which regulates bodily responses to sound. Moreover, the medial geniculate nucleus may play a significant role in sound localization and processing of complicated vocal communications (Calford & Aitkin, 1983; Rouiller et al., 1985). The suprageniculate nucleus belongs to the intermediate nuclei of the thalamus, where it makes up the caudal portion. The linkages indicate that this nucleus contributes in some manner to opto‐motor or auditivo‐motor processes (Droogleeverfortuyn & Minderhoud, 1965). Further, it is considered to be the region responsible for pain perception in an animal model (Schmahmann, 2003). Thus, this phenomenon of increase in thalamic nuclei volumes may be because of the continuous excitation of the cortex associated with visual, auditory, and sensory function as well as the stimulation of these thalamic nuclei. This may be also explained by an increase in the thalamic nuclei volumes for persistent relay of neuronal excitation from the occipital lobe to other brain regions. All these findings suggest an important role of the thalamus in the epileptic network of OLE.

Furthermore, we observed that the volumes of the right and left parafascicular nuclei, which are a part of the intralaminar group of the thalamus, were decreased in patients with OLE compared with those in healthy controls. The parafascicular nucleus is innervated by afferent fibers originating from the somatosensory and motor cortex, reticular thalamic nucleus, substantia nigra pars reticulata, mesencephalic reticular formation, and pedunculopontine nucleus, with glutamatergic projections to the motor, somatosensory, entorhinal cortex, striatum, and subthalamic nucleus (Brown et al., 2010; Feger et al., 1994). The parafascicular nucleus is involved in cognitive, sensory, and motor functions, as well as sensorimotor coordination, nociception, and arousal. In addition, the parafascicular nucleus of the thalamus is engaged in the generation of physiological oscillatory rhythms (Brown et al., 2010; Feger et al., 1994). The same finding was also observed in patients with temporal lobe epilepsy in a previous study, showing a decrease in the parafascicular nuclei volumes (Lee, Seo, & Park, 2020). In spite of epilepsies originating from different lobes, as inferred from the reduction in volume of the same nucleus in the thalamus, the parafascicular nucleus may be among the structures most vulnerable to seizure‐related damage or a structure commonly involved in the focal epilepsy network. The involvement of the parafascicular nucleus in the generation of physiological oscillatory rhythms also supports this assumption (Langlois et al., 2010). Although no studies have attempted to discover the associations between the parafascicular nucleus changes and OLE, a study investigated the relationship between the parafascicular nucleus and temporal lobe epilepsy. The study demonstrated the involvement of the parafascicular nucleus in the occurrence of hippocampal paroxysmal discharges in a chronic animal model of temporal lobe epilepsy in male mice (Langlois et al., 2010). Furthermore, the parafascicular nucleus is a part of the centromedian–parafascicular nuclear complex. Several clinical studies utilizing high‐frequency stimulations of the centromedian nucleus have already revealed a reduction in focal and generalized seizures in patients with epilepsy, attributable to the activations of ipsilateral desynchronizing thalamocortical projections (Son et al., 2016; Valentin et al., 2013). Therefore, in patients with OLE who do not respond well to ASMs, the parafascicular nucleus may be a target for neurostimulation. Further studies are needed to confirm these findings.

We found no alterations in the intrinsic thalamic network of patients with OLE compared with that of healthy controls. A plausible explanation for this finding is that the occipital cortex is the primary origin of epilepsy with little involvement of the thalamus, despite the thalamus playing an important role in the epileptic network of OLE. Another assumption is that this may be because of the compensation effects of the other thalamic nuclei when there are reduced volumes of some individual thalamic nuclei. Similar findings were reported in a study on patients with migraine, another neurological disease (Shin et al., 2019). The anteroventral and medial geniculate nuclei volumes were significantly increased, whereas the parafascicular nuclei volumes were decreased in patients with migraine compared with those in healthy controls. However, the network measures of the intrinsic thalamic network were not different between the two groups (Shin et al., 2019).

To our knowledge, this was the first study to focus on the thalamic changes in patients with OLE; however, it had some limitations. First, because the sample size was relatively small, we could not analyze the difference of the thalamic nuclei volumes according to side of epilepsy origin. However, OLE is a rare focal epilepsy, and only patients with well‐documented ictal rhythm on EEG and ictal semiology compatible with OLE were enrolled. In addition, we only included patients with newly diagnosed OLE to exclude the effects of ASMs on the thalamic nuclei volumes or intrinsic thalamic network. Second, this study was not a research based on pathology, but a study using brain MRI. Since the thalamic nucleus is a very small structure, it is difficult to rule out the possibility that an error may have occurred during segmentation. However, we were able to reduce the bias that occurs during manual segmentation by using the automatic segmentation method provided by the FreeSurfer program. This tool showed good agreement with previous histological studies of the thalamus in terms of volumes of representative nuclei, and revealed excellent test–retest reliability, robustness to changes in input MRI contrast, and ability to detect differential thalamic effects in individuals with neurological diseases (Iglesias et al., 2018).

## 5 CONCLUSION

We successfully demonstrated the alterations in the volumes of individual thalamic nuclei, especially the increased medial geniculate and suprageniculate, and decreased parafascicular nuclei volumes in patients with OLE compared with those of healthy controls despite no changes in the whole‐thalamic volumes. These findings suggest an important role of the thalamus in the epileptic network of OLE.

## AUTHOR CONTRIBUTIONS

Conception and design: Dong Ah Lee, Ho‐Joon Lee, and Kang Min Park. Acquisition of data: Ho‐Joon Lee and Kang Min Park. Analysis and interpretation of data: Ho‐Joon Lee and Kang Min Park. Drafting the manuscript or revising: Dong Ah Lee and Kang Min Park. Final approval: Kang Min Park.

## CONFLICT OF INTEREST STATEMENT

The authors declare no conflicts of interest.

### PEER REVIEW

The peer review history for this article is available at https://publons.com/publon/10.1002/brb3.2968.

## Data Availability

The data that support the findings of this study are available from the corresponding author upon reasonable request.
